# The relationship between ezrin and podoplanin expressions in keratocystic odontogenic tumors

**DOI:** 10.1186/1472-6831-14-150

**Published:** 2014-12-05

**Authors:** Denise Tostes Oliveira, Laís Priscila de Santis, Agnes Assao, Kellen Cristine Tjioe, Suely Nonogaki, José Roberto Pereira Lauris, Fernando Augusto Soares

**Affiliations:** Department of Stomatology, Area of Pathology, Bauru School of Dentistry, University of São Paulo, Alameda Octávio Pinheiro Brisolla, 9-75, 17012-901 Bauru São Paulo, Brazil; Adolfo Lutz Institute, Pathology Division, São Paulo, Brazil; Department of Community Dentistry, Bauru School of Dentistry, University of São Paulo, Bauru São Paulo, Brazil; Department of Pathology, A.C. Camargo Cancer Hospital, São Paulo, Brazil

**Keywords:** Keratocystic odontogenic tumor, Ezrin, Podoplanin

## Abstract

**Background:**

The aims of this study were to investigate the immunolocalization of ezrin and its relationship with the podoplanin expression in keratocystic odontogenic tumors.

**Material and Methods:**

The immunohistochemical expressions of ezrin and podoplanin by odontogenic epithelium were evaluated in keratocystic odontogenic tumors using monoclonal antibodies.

**Results:**

Our results showed strong cytoplasmic ezrin and membranous podoplanin expressions in basal epithelial layer of all keratocystic odontogenic tumors. The cytoplasmic and membranous ezrin expressions were also detected in suprabasal epithelial layers of tumors. Statistically significant difference between cellular immunolocalization of ezrin and podoplanin odontogenic epithelium were found by Wilcoxon’s test (p < 0.05). No correlation between both proteins in keratocystic odontogenic tumors was detected by Spearman test.

**Conclusions:**

These results suggest that ezrin and podoplanin may contribute to the expansive growth and local invasiveness of keratocystic odontogenic tumors. Additionally, as both proteins were overexpressed by odontogenic epithelium, their possible roles need to be further explored in benign odontogenic tumors.

## Background

Podoplanin expression has been detected in epithelial cells of developing tooth germ [1, 2] and in odontogenic epithelium of benign tumors [3–10].

The presence of podoplanin in human tooth germ tissues, adult teeth and cystic odontogenic lesions suggested that this protein probably is involved in mechanisms of cell adhesion, epithelial-mesenchymal transition and invasion, and expansive growth of cystic odontogenic lesions [5].

Previous studies conducted in our laboratory investigated the association of podoplanin with cellular proliferative activity, determined by Ki-67 antibody, in ameloblastomas [7] and keratocystic odontogenic tumors [9]. We did not find a statistically significant correlation in ameloblastomas, however this association was observed in keratocystic odontogenic tumors. Moreover, both podoplanin and Ki-67 expressions were stronger and co-localized in keratocystic odontogenic tumors when compared to the orthokeratinized odontogenic cysts, an indolent lesion. These fingings suggested that podoplanin positive cells are located in the cell proliferation centre indicating a role for this protein in the process of tumoral invasion [9].

Furthermore, Friedrich et al. [8] showed that podoplanin expression pattern is similar between keratocyst odontogenic tumor diagnosed in sporadic and in nevoid basal cell carcinoma syndrome and, the authors reinforced the probable association of this protein with invasion and local recurrences of the tumor.

Recent findings prove that podoplanin is important to drive directional cell migration in epithelial and tumor cells [11]. Then, the ability of podoplanin to remodel cytoskeleton and form filipodia-like membrane extension [12] has been suggested as important factor in movement of odontogenic epithelial cells [6]. This podoplanin-induced cell motility through of actin cytoskeleton rearrangement seems to be dependent on the interaction with the cytoplasmatic ezrin [13], a member of ERM (ezrin, radixin, moesin) protein family protein [14, 15].

The currently study was designed to analyze the immunolocalization of ezrin and its relationship with podoplanin expression in keratocystic odontogenic tumors. To the best of our knowledge, this is the first report of ezrin immunostaining in an odontogenic tumor.

## Methods

### Patients and tumor samples

All surgical specimens of keratocystic odontogenic tumor analyzed in this study were obtained from the Laboratory of Pathology, Bauru School of Dentistry, University of São Paulo, between 2002 and 2010. The inclusion criteria were: i) patients with diagnosis of keratocystic odontogenic tumor based on the classification of the World Health Organization [16], determined by the sum of the clinical, radiographic, and microscopic data; ii) availability of the paraffin block with sufficient and representative amount of odontogenic tumor for microscopic analysis. Applying the inclusion criteria, 18 keratocystic odontogenic tumors were selected for investigation of podoplanin and ezrin immunostaining. This study was approved by the Research Ethics Committee of the Bauru School of Dentistry, University of São Paulo (process #85612/2012).

### Immunohistochemistry

Formalin-fixed 3 μm sections of keratocystic odontogenic tumors were obtained from the pathology archive for immunohistochemistry analysis of the ezrin and podoplanin expressions by odontogenic epithelium. After antigen retrieval using 10 mM citrate buffer, pH 6.0, in a domestic pressure cooker (model Eterna 4½ L; Nigro, Araraquara, Brazil) for 4 min, endogenous peroxidase activity was blocked by incubation in 3% H_2_O_2_ for 20 min. Each section was incubated overnight at 48°C with the primary monoclonal anti-podoplanin antibody (D2-40 clone, code#3619-1; Dako North America, Inc., Carpinteria, CA, USA), dilution 1:200 or anti-ezrin antibody (Dako North America, Inc., CA, USA), dilution 1:1000, in phosphate-buffered saline (PBS) with bovine serum albumin (cat. #A2153, Sigma-Aldrich, St Louis, MO, USA) solution to block a non-specific reaction. Then, each section was incubated with Advance HRP Link System (cat.#4067, Dako North America, Carpinteria, CA, USA) for 30 min at 37°C. Both antibodies were detected using 3.30-diaminobenzedine tetrahydrochloride(DAB, cat. #D-5637, Sigma-Aldrich, St. Louis, MO, USA). Tumor sections were counterstained with Mayer’s hematoxylin before being dehydrated and cover slipped. Palatine tonsils and intestine were used as positive control for podoplanin and ezrin, respectively. For a negative control, the primary antibody was omitted during the immunohistochemical staining. The ezrin and podoplanin expressions by odontogenic epithelium of the 18 keratocystic odontogenic tumors were evaluated in ten microscopic fields digitally captured using an Axiocam camera (Axiocam MR3; Zeiss, Jena, Germany) attached to a light microscope and recorded by Axiovision software (Axiovision 4.7; Zeiss). A score for ezrin and podoplanin immunostainings expressed by odontogenic epithelium was based on: a) the intensity of the immunostaining in the epithelial odontogenic cells (0 = absent, 1 = weak, 2 = moderate, 3 = strong, and 4 = very strong) and (b) the percentage of positive odontogenic cells (0 = 0% positive cells, 1 = <25% positive cells, 2 = 25–50% positive cells, 3 = 50–75% positive cells, and 4 = >75% positive cells). The final immunostaining score was determined by the sum of (a) + (b). Final scores ranged from 0 to 8 (0 = absent, 1–4 = weak, and 5–8 = strong). The difference and the correlation between membranous or cytoplasmic expression of ezrin and podoplanin expressed in keratocystic odontogenic tumors were determined by Wilcoxon’s test and Spearman’s correlation coefficient, respectively. The level of significance was set at 5% for all tests.

## Results

### Clinical features

Our sample consisted in sixteen patients diagnosed with keratocystic odontogenic tumors (nine of them were women and seven men). Patient’s age ranged from 9 to 68 years old (mean 28 years) and they were predominantly white (75%). Most of the tumors were located at posterior region of the mandible (62.5%), followed by maxilla (25%), and in 3 cases the localization was not specified by the surgeon. From the total of 16 patients with keratocystic odontogenic tumors, 4 of them had Gorlin-Goltz syndrome and from these, 2 had multiple lesions but in different locals and periods.

### Podoplanin expression by keratocystic odontogenic tumors

**Figure 1:**
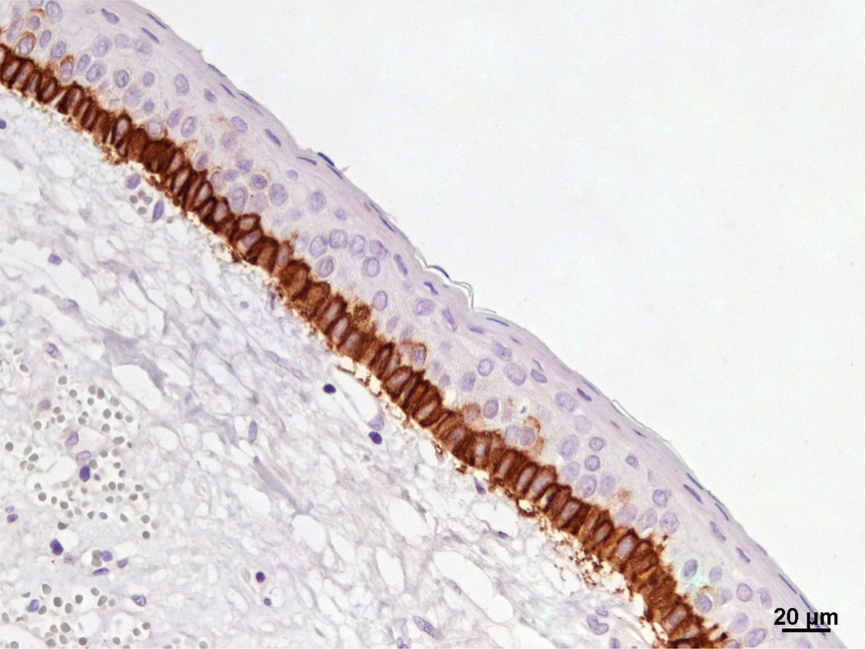
**Expression of podoplanin in keratocystic odontogenic tumor.** Positivity restricted to the basal layer (IHQ, 400×).

The basal layer of the normal oral mucosa presented positivity for membranous podoplanin as well as the lymphatic vessels in the subjacent connective tissue, confirming the integrity of the immunohistochemical reaction. The membranous and cytoplasmic expression of podoplanin was intensively concentrated at the basal layer of the epithelium in all keratocystic odontogenic tumors (Table 1). The upper layers of epithelium were negative for this protein (Figure 1) and peripheral cells of daughter cysts strongly expressed this protein.

**Table 1:** **Membranous and cytoplasmic podoplanin expression in 18 keratocystic odontogenic tumors**

	PODOPLANIN				
	Membranous		Cytoplasmic		p*
	N	%	N	%	
**Absent**	0	0	0	0	1.000
**Weak**	0	0	1	5.56	
**Strong**	18	100	17	94.44	
**TOTAL**	**18**	**100**	**18**	**100**	

Bauru School of Dentistry – University of São Paulo, São Paulo, 2002 to 2010.

*Wilcoxon test; ρ = statistical significance level of 0.05.

### Ezrin expression by keratocystic odontogenic tumors

**Figure 2:**
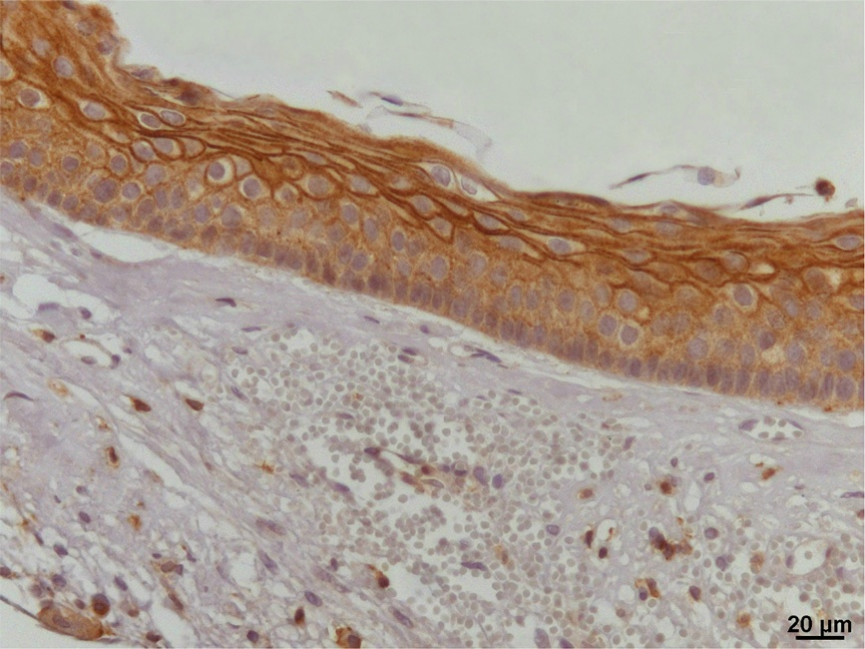
**Expression of ezrin in keratocystic odontogenic tumor.** Note cytoplasmic positivity in basal and supra-basal layers and membranous expression in the most superficial level of the odontogenic epithelium (IHQ, 400×).

Ezrin was strongly expressed in the cytoplasmic membrane of the spinous layer of normal oral mucosa. Most of keratocyst odontogenic tumors epithelial cells (83.33% of cases) strongly expressed cytoplasmic ezrin (Table 2). The cytoplasmic ezrin was frequently detected in epithelial basal layer and higher membranous ezrin positivity could be noted in supra-basal layers of epithelium. The absent or weak membranous ezrin expression by epithelial cells was observed in approximately 61% of the tumors. The odontogenic epithelial cells of daughter cysts also showed cytoplasmic and membranous ezrin (Figure 2).

**Table 2:** **Membranous and cytoplasmic ezrin expression in 18 keratocystic odontogenic tumors**

	EZRIN				
	Membranous		Cytoplasmic		p*
	N	%	N	%	
**Absent**	10	55.56	2	11.11	0.007
**Weak**	1	5.56	1	5.56	
**Strong**	7	38.88	15	83.33	
**TOTAL**	**18**	**100**	**18**	**100**	

Bauru School of Dentistry – University of São Paulo, São Paulo, 2002 to 2010.

*Wilcoxon test; ρ = statistical significance level of 0.05.

### Relationship between ezrin and podoplanin expressions in keratocystic odontogenic tumors

The Wilcoxon’s test was used to compare the subcellular expression of ezrin and podoplanin in benign odontogenic tumors. The differences among membranous ezrin and membranous (p = 0.003) or cytoplasmic (p = 0.003) podoplanin expressions were statistically significant (Table 3). Furthermore, there were no statistically significant differences between cytoplasmic ezrin and membranous (p = 0.109) or cytoplasmic (p = 0.201) podoplanin expressions, as llustrated in Table 3. The Spearman test did not demonstrate statistically significant correlation between membranous or cytoplasmic podoplanin and ezrin expressions in keratocystic odontogenic tumors (Table 4).

**Table 3:** **Comparison between membranous and cytoplasmic podoplanin and ezrin expression in 18 keratocystic odontogenic tumors**

	Podoplanin		Ezrin					
	M (%)	C (%)	M (%)	C (%)	M PO	M PO	C PO	C PO
					X	X	X	X
					M EZ	C EZ	M EZ	C EZ
**Absent**	0	0	55.56	11.11	**p = 0.003**	**p = 0.109**	**p = 0.003**	**p = 0.201**
**Weak**	0	5.56	5.56	5.56				
**Strong**	100	94.44	38.88	83.33				
**TOTAL**	**100**	**100**	**100**	**100**				

Bauru School of Dentistry – University of São Paulo, São Paulo, 2002 to 2010.

Wilcoxon test; ρ = statistical significance level of 0.05. PO: podoplanin; EZ: ezrin; M: Membranous; C: Cytoplasmic.

**Table 4:** **Membranous and cytoplasmic correlation between podoplanin and ezrin expression in 18 keratocystic odontogenic tumors**

	Membranous	Cytoplasmic
	Podoplanin	Podoplanin
**Membranous ezrin**	r = 0.000	r = −0.293
	p = 1.000	p = 0.293
**Cytoplasmic ezrin**	r = 0.000	r = −0.108
	p = 1.000	p = 0.670

Bauru School of Dentistry – University of São Paulo, São Paulo, 2002 to 2010.

r = Spearman’s rank correlation coefficient; ρ = statistical significance level of 0.05.

## Discussion

In recent years, the interest in podoplanin expression in benign odontogenic tumors has considerably increased [3, 4, 6–10, 17]. It has been suggested that this protein participates in the movement of odontogenic epithelial cells through actin cytoskeleton rearrangement [6, 9]. Although the odontogenic cell motility induced by podoplanin may be dependent on its interaction with the activated ezrin, the connection between both proteins has not previously been studied in odontogenic tumors.

Our results regarding the immunolocalization of podoplanin showed strong membranous and cytoplasmic expressions of the protein detected mainly in the basal layer of the odontogenic epithelium, even in daughter cysts. In normal oral mucosa, podoplanin expression was membranous and concentrated at the basal epithelial layer. Our findings corroborated those of previous authors’ [4, 8–10, 17].

Since Okamoto et al [4] proposed, for the first time, a possible role of the podoplanin in tumor invasiveness of keratocystic odontogenic tumors, further investigations have been conducted in order to understand the exact function of this protein in the pathogenesis and growth of these benign tumors [5, 8–10, 17]. Tsuneki et al. [17] verified that podoplanin positive cells are located in the cellular proliferative center of keratocystic odontogenic tumors suggesting that this protein acts as one of the key regulators in the proliferative process of this tumor. Similarly, the participation of podoplanin in local invasion of odontogenic tumors was reinforced by our previous findings [9]. We have demonstrated the correlation between the podoplanin expression and the cellular proliferation index in keratocystic odontogenic tumors [9]. Furthermore, an interesting study of Zhang et al [10] showed that in keratocystic odontogenic tumors, conservatively treated by decompression, there was a significant loss or reduction of podoplanin expression in the odontogenic epithelium. In radicular and folicullar cysts, Zustin et al [5] found podoplanin expression more homogenous, diffused at cytoplasm and membrane, in epithelial basal and parabasal layer. As podoplanin is correlated with other proteins involved in cell proliferation and tumor invasion [9, 17], its downregulation may be due to limited activity of the odontogenic epithelium after the surgical approach [10].

Despite the fact that the exact function of podoplanin in odontogenic tumors is poorly understood and largely unknown, the results of the present study taken together with our previous investigation [9] and those of others authors [5, 17, 8, 10] strongly suggest that this protein is involved in local invasion and in the expansive growth of keratocystic odontogenic tumors, probably orchestrating the odontogenic epithelial cytoskeleton activity.

A possible mechanism suggested is that podoplanin binds ezrin and, as a downstream, follows the phosphorylation of GTPase-RhoA [7]. This signaling pathway would promote the rearrangement of the actin cytoskeleton and induce the formation of filopodia-like projections, promoting the cellular movement [13]. So, in order to verify if podoplanin and ezrin are co-localized in keratocystic odontogenic tumors, we designed the present study.

Ezrin, an ERM protein family member, exerts primordial functions related to the regulation of cellular movement and structure by transducing signals between the plasma membrane and the actin cytoskeleton [14]. Ezrin is essential for many physiological cellular processes including regulation of actin cytoskeleton, maintenance of cell shape, adhesion, motility and modulation of signal transduction pathways [14].

In head and neck squamous cell carcinomas, ezrin was identified as a key molecule in tumor progression and metastatic behavior of neoplastic cells and it has been correlated with poor outcome [18–21], similarly to podoplanin [22]. However, to the best of our knowledge, this is the first evidence that confirmed the immunoexpression of ezrin in benign keratocystic odontogenic tumors.

The results presented here showed absence of membranous ezrin in 55.56% of keratocystic odontogenic tumors and, when present, this protein was mainly observed in the membrane of the non-proliferative supra-basal layers (Figure 2). From that we can hypothesize that ezrin may be promoting the cellular adhesion, as similarly observed in non-proliferating squamous cells of the normal human oral mucosa [18, 20, 21].

Furthermore, most of the odontogenic tumors (83.33%) showed strong cytoplasmic ezrin expression especially in the basal layer of odontogenic epithelium. Interestingly, the cytoplasmic ezrin reflects its activated [18, 20, 21] form and, in oral cancer, this pattern of cytoplasmic ezrin has been associated with invasive phenotype and more aggressive behavior of malignant cells [18–21]. These findings suggest that activated ezrin is necessary for the proliferation and/or invasion of odontogenic cells once its cytoplasmic positivity was mainly found in the basal layers of keratocystic odontogenic tumors, the region responsible for the tumor growth.

The distribution of ezrin in odontogenic keratocystic tumors was more heterogeneous than that of podoplanin (Tables 1 and 2) and statistically significant differences were observed among the localization of membranous ezrin and cytoplasmic or membranous podoplanin (Table 3). It was previously reported that ezrin is markedly phosphorylated in the presence of podoplanin overexpression [13]. This interaction of podoplanin and ezrin is responsible for many of the effects on the cytoskeleton, contributing to increased cell motility [13]. Additionally, there is evidence that ERM function in organizing the cytoskeleton is mediated through its ability to bind actin and to regulate GTPase-RhoA phosphorylation [13]. Interestingly, the keratocystic odontogenic tumors presented strong membranous podoplanin and cytoplasmic ezrin expression, which correspond to the activated forms of both proteins. The podoplanin activated form (membranous) was mostly found in the basal layer, indicating a co-localization of active-ezrin and active-podoplanin in the highly active areas of keratocystic odontogenic tumors. However, this correlation was not statistically significant probably owing to the low number of negative tumors (0 for membranous podoplanin and 2 for cytoplasmic ezrin), which does not permit to establishing an associative relationship (Table 4).

## Conclusions

In summary, we first report that ezrin presents strong membranous expression in the supra-basal layer and strong cytoplasmic expression in the basal layer of keratocystic odontogenic tumors. Thus, our investigation suggests a role for ezrin and podoplanin in keratocystic odontogenic tumors and their molecular interactions need to be further explored in these kinds of tumors.

### Consent

This study has been approved by the Committee of ethics and research from Bauru School of Dentistry – University of São Paulo. CAAE: #03924312.0.0000.5417.

## Abbreviations

ERM: Ezrin, radixin and moesin
GTP-ase: Guanosine- triphosphatase.
